# Syndrome de Stewart Treves: une complication redoutable du lymphœdème

**DOI:** 10.11604/pamj.2016.25.89.8150

**Published:** 2016-10-17

**Authors:** Wafaa Labbardi, Fouzia Hali

**Affiliations:** 1 Service de Dermatologie Vénéréologie, CHU Ibn Rochd, Université Hassan II, Casablanca, Maroc

**Keywords:** Angiosarcome, lymphœdèsme, chirurgie, prévention, Angiosarcoma, lymphedema, surgery, prevention

## Image en médecine

**Figure 1:**
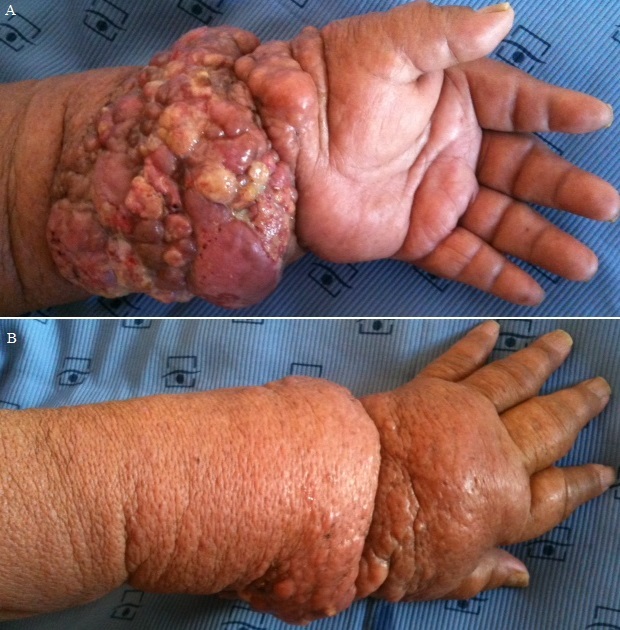
A) masse tumorale bourgeonnante polylobée d’allure angiomateuse sur lymphœdème du membre supérieur gauche (vue antérieure); B) masse tumorale bourgeonnante polylobée d’allure angiomateuse sur lymphœdème du membre supérieur gauche (vue postérieure)

Le syndrome de stewart-treves (SST) est une complication rare et gravissime du lymphœdème chronique des extrémités à type d’angiosarcome. Le diagnostic repose sur l’histologie et l’immuno-histochimie. Le traitement est essentiellement chirurgical. Ce syndrome survient dans 90% des cas dans un contexte de chirurgie mammaire. Cependant, ce contexte peut parfois manquer comme illustré dans notre observation. Le pronostic du STT est sombre avec une survie à cinq ans avoisinant les 10% d’où l’intérêt d’un diagnostic précoce et de la prévention des lymphœdèmes. Nous rapportons le cas d’une patiente âgée de 70 ans, sans antécédents de néoplasie mammaire, de chirurgie axillaire ou de radiothérapie qui présentait un œdème du membre supérieur gauche depuis 2010, compliqué deux ans plus tard par l’apparition d’une lésion nodulaire augmentant progressivement de taille. L’examen clinique retrouvait une masse tumorale bourgeonnante polylobée circonférentielle de l’avant-bras sur lymphœdème du membre supérieur gauche (Figure 1 A et Figure 1 B). Les aires ganglionnaires étaient libres. Le reste de l’examen somatique était normal. L’histologie objectivait une prolifération tumorale composée d’ébauches de cavités vasculaires bordées d’une couche de cellules endothéliales bien délimitée par une membrane basale et cernée par des péricytes avec expression du CD31 à l’immuno-histochimie. L’angioscanner du membre supérieur gauche montrait une masse tumorale de l’avant bras gauche avec compression vasculaire sans atteinte osseuse. Après une concertation pluridisciplinaire, une amputation mi-bras gauche était faite suivie d’une radiothérapie. L’évolution n’a pas noté de récidive. Le recul actuel est de 27 mois.

